# Catecholaminergic Contributions to Inhibitory Control Following Physical Fatigue: Behavioral and Neurophysiological Findings

**DOI:** 10.1002/ejsc.70231

**Published:** 2026-08-17

**Authors:** Y. Laurisa Arenales Arauz, Nina Omejc, Maria Alejandra Diaz, Jelle Habay, Bram Vanderborght, Kevin De Pauw, Bart Roelands, Uros Marusic

**Affiliations:** ^1^ Human Physiology and Sports Physiotherapy Research Group Faculty of Physical Education and Physiotherapy Vrije Universiteit Brussel Ixelles Belgium; ^2^ Department of Knowledge Technologies Jožef Stefan Institute Ljubljana Slovenia; ^3^ Jožef Stefan International Postgraduate School Ljubljana Slovenia; ^4^ BruBotics Vrije Universiteit Brussel and Imec Brussels Belgium; ^5^ Vital Signs and Performance Monitoring Research Unit LIFE Department Royal Military Academy Brussels Belgium; ^6^ Research Foundation Flanders (FWO) Brussels Belgium; ^7^ Institute for Kinesiology Research Science and Research Centre Koper Koper Slovenia; ^8^ Department of Health Sciences Alma Mater Europaea University Maribor Slovenia

**Keywords:** brain neurotransmission, cognitive control, EEG, event‐related potentials, physical fatigue

## Abstract

Acute physical fatigue can impair cognitive control, yet its underlying neurochemical mechanisms remain unclear. This study investigated whether catecholaminergic modulation influences behavioral and neural markers of inhibitory control following physical fatigue. Eighteen healthy, recreationally active adults (9 males, 9 females; 23.4 ± 2.2 years) completed a randomized, triple‐blind, placebo‐controlled crossover study. On separate visits, participants received methylphenidate (MPH; 20 mg; a dopamine and noradrenaline reuptake inhibitor), reboxetine (REB; 8 mg; a noradrenaline reuptake inhibitor), or placebo. Physical fatigue was induced by repeated bilateral leg extensions to task failure. Cognitive performance was assessed before and after physical fatigue using a Go/No‐Go task with electroencephalographic recording. Behavioral outcomes included reaction time and accuracy, while event‐related potentials measured neural stages of response execution and inhibition (N2 and P3). Mixed‐effects models were used for statistical analysis. For No‐Go trials, a significant MPH × Time interaction was observed for accuracy (*p* = 0.008), with improved post‐fatigue performance following MPH administration (*p* = 0.048). At the neural level, MPH was associated with shorter fronto‐central No‐Go N2 latency (*p* = 0.038) and altered fatigue‐related changes in No‐Go P3 latency (*p* = 0.047). REB did not produce comparable behavioral or neural effects. These findings provide pharmacological evidence that catecholaminergic mechanisms contribute to inhibitory control following physical fatigue. The differential effects of MPH and REB suggest that selective noradrenergic enhancement alone is insufficient to maintain inhibitory control following physical fatigue. Instead, the findings implicate broader dopaminergic and noradrenergic mechanisms, potentially involving alterations in the temporal dynamics of inhibitory processing.

**Trial Registration:**

(G095422N and identifier NCT05880342)

## Highlights

- Methylphenidate improved inhibitory performance following fatigue, potentially through altered temporal dynamics
- Reboxetine did not affect behavioral or neural markers of inhibitory control
- Differential drug effects suggest that both dopaminergic and noradrenergic mechanisms contribute to inhibitory control following physical fatigue

## 1 Introduction

Acute physical fatigue frequently occurs in sport and physically demanding occupations and is characterized by reduced motor performance and increased perception of effort (Behrens et al. 2022). Physical fatigue arises from the dynamic interplay of peripheral and central processes (Behrens et al. 2022; Enoka and Duchateau 2016; Meeusen et al. 2021) and its effects extend beyond motor performance to cognitive function. Physical fatigue can be associated with slower responses and reduced accuracy during cognitive tasks (Skala and Zemková 2022; Yao et al. 2025), although effects vary across tasks and contexts (Yao et al. 2025; Sudo et al. 2017). Recent frameworks further emphasize that fatigue‐related cognitive changes may not reflect a global decline in performance, but rather a reallocation of neural resources depending on task demands and motivational context (Boksem and Tops 2008; Marcora 2009; Dietrich and Audiffren 2011). This perspective suggests that preserved or even improved behavioral performance after physical fatigue may occur alongside altered neural processing strategies, highlighting the importance of integrating behavioral and neurophysiological measures. The role of central catecholaminergic systems in shaping these changes remains unclear, yet understanding this role is crucial for determining whether cognitive performance is maintained or compromised.

Physical fatigue has been shown to influence higher‐order executive functions such as attention, decision‐making and response inhibition (Skala and Zemková 2022; Moore et al. 2012). Response inhibition refers to the ability to withhold automatic responses when they conflict with current task demands (Diamond 2013). Despite its frequent assessment using Go/No‐Go, Stroop, and flanker paradigms, evidence for physical fatigue‐related effects on inhibitory control remains mixed. After simulated operational exhaustion, a marked reduction in No‐Go accuracy was observed (Zhao et al. 2024). Similarly, following exhaustive cycling protocols, task accuracy decreased in high‐conflict Stroop trials (Yao et al. 2025; Itagi et al. 2018). In contrast, other studies report faster response times without changes in accuracy in a flanker task (Finkenzeller et al. 2018) and stable Go/No‐Go accuracy (Sudo et al. 2017). Together, these findings suggest that behavioral measures alone may be insufficient to clarify how acute physical fatigue alters inhibitory control.

Neurophysiological measures such as electroencephalography (EEG) may help clarify how acute physical fatigue alters the neural mechanisms underlying inhibitory control (Falkenstein et al. 1999; Huster et al. 2013). Event‐related potentials (ERPs) allow the separation of distinct processing stages underlying response inhibition with millisecond precision (Falkenstein et al. 1999). In inhibitory control paradigms, the N2 component is commonly associated with conflict monitoring and early control processes, whereas the P3 component reflects later stages of response evaluation and the implementation of inhibition (Falkenstein et al. 1999; Smith et al. 2006). Physical fatigue has been shown to modulate these components. Reduced P3 amplitude has been consistently reported in cognitive tasks following exhaustion (Yao et al. 2025; Zhao et al. 2024; Itagi et al. 2018), indicating reduced neural resource allocation and conflict resolution during inhibitory processing (Yao et al. 2025). Shortened P3 latency has also been observed (Finkenzeller et al. 2018), suggesting accelerated stimulus evaluation. In contrast, findings for the earlier N2 component are less consistent, with increased amplitudes (Zhao et al. 2024) and reduced amplitudes (Finkenzeller et al. 2018) reported. These findings suggest stage‐specific effects of physical fatigue on inhibitory processing. The complexity of these effects underscores the need to identify potential mediators, with central neuromodulation emerging as one plausible mechanism (McMorris et al. 2011; Sanders 1983).

In cognitive‐energetic models (McMorris et al. 2011; Sanders 1983), a non‐linear relationship (inverted‐U) between exercise intensity and executive performance has been proposed. This framework suggests that optimal exercise intensity is associated with levels of central neuromodulatory activity that support cognitive control. In contrast, insufficient or excessive activation, such as during physical fatigue, may impair processing efficiency (McMorris et al. 2011). Dopamine (DA) and noradrenaline (NA) are considered key neuromodulators within this framework, given their roles in regulating arousal, attention, and motivation (Ranjbar‐Slamloo and Fazlali 2020). Evidence from pharmacological studies using reuptake inhibitors further indicates a central catecholaminergic contribution to physical fatigue and performance (Zheng and Hasegawa 2017). Selective NA reuptake inhibition generally reduced physical performance (Klass et al. 2012; Roelands, Goekint, et al. 2008), whereas dual DA/NA reuptake inhibition only demonstrated ergogenic effects under heat stress (Zheng and Hasegawa 2017; Roelands, Goekint, et al. 2008; Roelands, Hasegawa, et al. 2008; Roelands and Meeusen 2010; Meeusen and Roelands 2010; Roelands et al. 2012; Arauz et al. 2026). In a previously published report from the current randomized, triple‐blind crossover trial (Arauz et al. 2026), we examined performance and perceptual outcomes following administration of methylphenidate (MPH; a DA/NA reuptake inhibitor) and reboxetine (REB; a NA reuptake inhibitor) prior to dynamic leg extensions to exhaustion. MPH increased alertness, mood, and perceived performance without improving physical performance, whereas REB reduced performance without affecting perception. Whether these dissociable catecholaminergic effects extend to cognitive control and its neural dynamics remains unclear.

To date, no studies have examined whether catecholaminergic modulation influences inhibitory control following localized muscle exhaustion or the neural processes underlying such effects. To address this gap, we combined pharmacological manipulation with behavioral and EEG measures of inhibitory control before and after physical fatigue. Our primary objective was to determine whether these pharmacological manipulations influenced behavioral inhibitory performance. As a secondary objective, we examined whether fatigue‐related changes in behavioral performance were accompanied by corresponding changes in the canonical N2 and P3 ERP components during inhibitory (No‐Go) processing. Go‐trial results were included to determine whether pharmacological effects were specific to inhibitory control or reflected broader alterations in response execution. Given its role in prefrontal cognitive control (Zheng and Hasegawa 2017; Dockree et al. 2017), we hypothesized that MPH would reduce the negative effects of physical fatigue on inhibitory performance and potentially modulate ERP components associated with inhibitory processing.

## 2 Materials and Methods

### 2.1 Ethics, Registration and Reporting

This study was approved by the Medical Ethics Committee of VUB and UZ Brussel (protocol number: 2022‐002836‐30) and conducted in accordance with the Declaration of Helsinki. Additional approval was obtained from the Federal Agency for Medicines and Health Products (reference number: 1311860). The trial was registered on clinicaltrials.gov (NCT05880342) and adhered to the SPIRIT guidelines (Chan et al. 2013). The present manuscript reports the cognitive and neurophysiological outcomes, whereas exercise performance and perceptual outcomes have been reported previously (Arauz et al. 2026). EEG acquisition, preprocessing, and reporting followed current consensus recommendations for EEG and ERP research (Keil et al. 2014; Pernet et al. 2019). Reporting of the randomized crossover design was guided by the updated CONSORT 2025 statement (Hopewell et al. 2025) and the statistical assessment checklist (Mansournia et al. 2021). All participants provided written informed consent prior to participation.

### 2.2 Participants

In total, 18 participants (9 males, 9 females; age 23.4 ± 2.2 years; height: 170.0 ± 9.4 cm; body mass: 66.1 ± 9.9 kg; BMI 22.9 ± 2.6 kg/m^2^; fat percentage 20.8 ± 8.5%) were included in this study. Participants were required to engage in recreational physical activity at least once per week and to be free from neurological, psychiatric, or chronic medical conditions. Individuals with competitive or elite athletic training backgrounds were excluded to minimize confounding effects of advanced physical conditioning. Additional exclusion criteria included pregnancy, recent musculoskeletal injury, medications affecting the central nervous system, known allergies to study substances, and participation in other research studies or medical treatments during the study period.

### 2.3 Study Design and Setting

This study used a randomized, triple‐blind, counterbalanced, within‐subject crossover design to investigate the effects of acute physical fatigue and pharmacological manipulation on cognitive performance and neural responses. Following one familiarization session, each participant completed three experimental sessions under different pharmacological conditions: MPH, REB, and placebo. Experimental sessions were separated by 1–2 weeks and conducted during the morning (07:00–12:00) to minimize potential carry‐over effects and ensure standardized testing conditions. All testing sessions were conducted in a sound‐insulated laboratory by at least two trained researchers, with medical supervision available throughout.

### 2.4 Pharmacological Manipulation and Blinding

In each experimental session, participants received two identical‐looking tablets providing a total dose of either 20 mg MPH, 8 mg REB, or placebo (P‐Tabletten weiß 7 mm Lichtenstein, lactose‐based tablets). MPH acts as a DA and NA reuptake inhibitor, whereas REB selectively inhibits NA reuptake, thereby increasing extracellular monoamine availability (Zheng and Hasegawa 2017). Treatment order was counterbalanced using a Latin square design. All study medication was prepared, coded, and dispensed by the hospital pharmacy. Participants and investigators remained blinded to treatment allocation throughout data collection and analysis. Unblinding occurred only after completion of all statistical analyses.

### 2.5 Experimental Procedures

#### 2.5.1 Familiarization Session

Prior to experimental testing, participants attended a familiarization session that included medical screening, explanation of all procedures, and practice of both the physical fatigue protocol and the cognitive task. This session ensured task comprehension, minimized learning effects, and confirmed eligibility for participation. During the familiarization session, an estimate of the one‐repetition maximum for the leg extension exercise was obtained to determine individualized loading for the physical fatigue protocol (Arauz et al. 2026).

#### 2.5.2 Experimental Session

Upon arrival, participants completed a pretest checklist and ingested the assigned study medication (placebo, MPH, or REB). Thirty minutes later, they consumed a standardized breakfast (620 kcal; 117 g of carbohydrates, 26 g of protein, 3.05 g of fat). EEG cap placement and electrode preparation, including the application of conductive gel, were then performed and required approximately 30–45 minutes. Approximately 90 minutes after drug ingestion, participants performed the baseline Go/No‐Go task with EEG recording. This timing corresponded to near‐peak plasma levels of MPH (Dockree et al. 2017) and near peak plasma levels of REB (Wasik and Siwek 2019). Physical fatigue was induced using repeated bilateral leg extensions to task failure at an individualized load corresponding to 60% of one‐repetition maximum. The effectiveness of the protocol in inducing exhaustion was previously established (Arauz et al. 2026) and is briefly summarized as a manipulation check in the Results. Immediately following the leg extensions, participants repeated the Go/No‐Go task with EEG acquisition to assess acute physical fatigue‐related effects on cognition.

### 2.6 Cognitive Task

**FIGURE 1:**
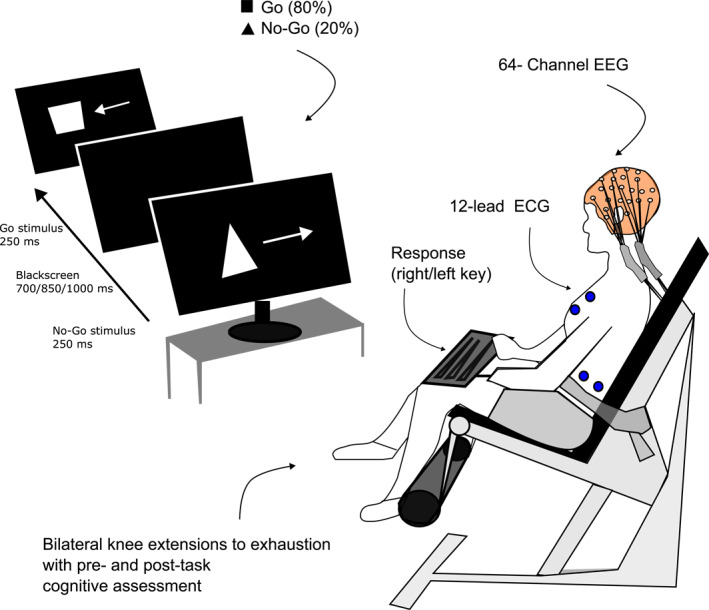
Experimental task and setup participants completed an 8‐min visual Go/No‐Go task (Go: 80%, No‐Go: 20%; stimulus duration: 250 ms; inter‐stimulus interval: 700/850/1000 ms) before and after a bilateral knee extension protocol performed to exhaustion. Participants were seated and securely strapped to the leg extension device to ensure body stabilization. EEG was recorded using a 64‐channel system.

Figure 1 presents the experimental task and set‐up. Cognitive performance was assessed using a computerized Go/No‐Go task programmed in E‐prime 3.0 (Psychology Software Tools, Pittsburgh, PA, USA). The task consisted of 420 trials, including 336 Go trials (80%) and 84 No‐Go trials (20%). The stimuli were presented for 250 ms and separated by an interstimulus interval of 700, 850 or 1000 ms in randomized order. This resulted in a total task duration of approximately 8 minutes. Participants were seated in a fixed position at an approximate viewing distance of 100 cm from the display monitor. Each trial consisted of the central presentation of a white geometric figure (square or triangle; area 42 cm^2^) and a directional arrow (left or right; 6.5 cm) on a black background. Participants responded to the arrow direction (Go: square) or withheld responses (No‐Go: triangle), as quickly and accurately as possible. Behavioral data were recorded using E‐Prime, including reaction times for correct Go trials and accuracy for both Go and No‐Go trials. These measures assessed attentional processing and response inhibition.

### 2.7 EEG Acquisition and Preprocessing

Continuous EEG was recorded using a wireless amplifier (LiveAmp, Brain Products GmbH, Munich, Germany) with 64 active Ag/AgCl electrodes mounted on an actiCAP slim/snap cap and connected via a LiveAmp actiCAP adapter. Electrodes were positioned according to the international 10–20 system. During recording, FCz served as the reference and AFz as the ground electrode. The signal was sampled at 500 Hz using BrainVision Recorder software (Brain Products GmbH, Munich, Germany), and electrode impedances were maintained below 15 kΩ. Event markers corresponding to stimulus onset and behavioral responses were generated using E‐Prime 3.0 (Psychology Software Tools, Pittsburgh, PA). These markers were transmitted via a hardware trigger box to the EEG acquisition system to ensure temporal synchronization between behavioral and electrophysiological data.

**FIGURE 2:**
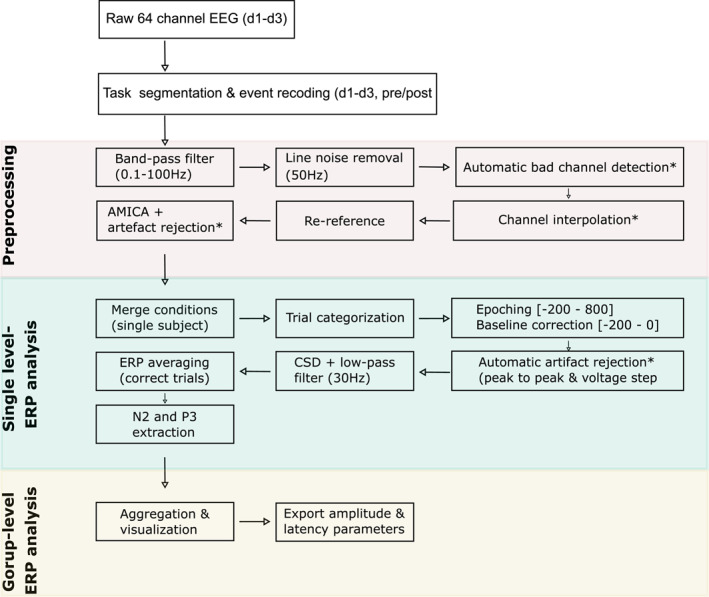
EEG preprocessing and ERP analysis pipeline * Indicated when additional visual inspection occurred of the automated processes raw 64‐channel EEG data are segmented and preprocessed using standard filtering, noise removal, channel quality control, and referencing procedures. Cleaned data are then organized into trials and subjected to single‐subject ERP analysis, including epoching, baseline correction, and artifact handling, followed by extraction of relevant components. Finally, group‐level aggregation and visualization are performed, with export of amplitude and latency measures for statistical analysis.

A schematic overview of the EEG data analysis pipeline is presented in Figure 2. The preprocessing pipeline (similar to those of Marusic et al. (2022) and Omejc et al. (2025)) was performed in MATLAB (Version 2022b; The MathWorks, USA) using the EEGLAB toolbox (Delorme and Makeig 2004). The approach aligned with current recommendations for EEG data recorded under movement conditions, particularly regarding artifact removal (Gorjan et al. 2022). Preprocessing was performed separately for each pharmacological condition. Continuous recordings were restricted to Go/No‐Go task segments based on experimental markers, retaining a short pre‐task buffer (200 ms) to allow for baseline correction (duration of each dataset: approximately 16 minutes). Event codes were recoded to uniquely identify pharmacological condition (1, 2, or 3) and time (pre/post). Data were filtered using 0.1 Hz high‐pass and 100 Hz low‐pass filter (Hamming windowed sinc FIR filters), and 50 Hz line noise was removed using the ZapLine plus method (Klug and Kloosterman 2022; de Cheveigné 2020). Bad channels were automatically identified using the Clean Rawdata EEGLAB plugin (flatline criterion = 10 s, channel correlation criterion = 0.85, maximum bad time = 0.5), manually inspected, and replaced using spherical spline interpolation. On average, 7.0 ± 3.24 of the 64 electrodes were interpolated. Data were subsequently re‐referenced to the average reference. After re‐referencing, Adaptive Mixture Independent Component Analysis (AMICA; 2000 iterations) was employed, and independent components were classified using the ICLabel plugin (Pion‐Tonachini et al. 2019). Components classified as “eye” artifacts were removed when classification confidence exceeded 0.80, and components classified as muscle, cardiac, line noise, or channel noise were removed when confidence exceeded 0.85. The cleaned continuous datasets were saved for subsequent ERP segmentation and analysis. All components were visually inspected, and additional artifact components were removed when they clearly reflected non‐neural activity. On average, 6.6 ± 3.79 independent components were removed from 49.3 ± 6.69 total components, corresponding to 14% of components.

### 2.8 ERP Analysis and Extraction

ERP analyses were performed in MATLAB using EEGLAB and ERPLAB (Lopez‐Calderon and Luck 2014). For each participant, the cleaned continuous datasets from the three pharmacological conditions were concatenated into a single file (single‐subject ERPs). ERPLAB event list files were created, and events were assigned to bins using a Binlister descriptor file. EEG data were epoched from −200 to 800 ms relative to stimulus onset and baseline‐corrected using the [−200 0] ms interval. Artifact rejection was performed on epoched data using combined peak‐to‐peak amplitude and voltage‐step criteria across all channels and the full epoch window. Thresholds were adaptively adjusted to prevent excessive data loss, such that no more than 5% of trials were rejected based on peak‐to‐peak amplitude and no more than 10% based on voltage‐step criteria. Automatically flagged epochs were visually inspected and manually corrected when required. Current source density transformation (Luck 2014) was applied to the epoched data and ERPs were computed by averaging artifact‐free trials within each bin. ERP waveforms were additionally low‐pass filtered at 30 Hz (6th order Butterworth, zero‐phase). Trials were categorized according to stimulus type and response accuracy using ERPLAB bin definitions. Only correct trials were retained, and ERP averages were computed separately for Go trials and No‐Go trials, further stratified by pharmacological condition (d1–d3) and time (pre/post). Electrode clusters were selected based on canonical Go/No‐Go ERP topographies (Huster et al. 2013). Given that inhibitory‐control‐related N2 and P3 components are typically observed over fronto‐central scalp regions (Hervault et al. 2025), a fronto‐central cluster (FC1, FC2) was selected as the primary region of interest. A centro‐parietal cluster (CPz, CP3, CP4) was additionally included to examine the spatial distribution of ERP effects and to capture the canonical scalp distribution of the Go P3 component (Hervault et al. 2025). Subsequently, ERP component amplitudes and latencies were automatically extracted within predefined time windows (N2: 200–350 ms; P3: 300–500 ms) for each participant and condition (Falkenstein et al. 1999; Huster et al. 2013). Individual ERP waveforms were visually inspected to verify correct peak identification. The resulting ERP datasets and parameter tables were used for subsequent group‐level statistical analyses in RStudio.

### 2.9 Statistical Analyses

#### 2.9.1 Sample Size Determination

Sample size was determined a priori using simulation‐based power analyses (Green and MacLeod 2016) for the planned drug × time effects. Simulations were conducted using linear mixed‐effects models (with a random intercept for each participant), with variance estimates and effect sizes informed by prior work (Warren et al. 2023). Specifically, the planning model assumed a small to moderate MPH‐specific drug × time interaction (0.25 μV) relative to placebo and a minimal REB interaction (0.05 μV). Based on 500 simulations (*α* = 0.05), *N* = 18 yielded an estimated power of 82% to detect the drug × time interaction for P3 No‐Go amplitude.

#### 2.9.2 Data Preparation

Data preparation was performed in Rstudio version 4.2.2 (R Core Team). Data were reorganized into long format prior to analysis. Experimental factors were coded as within‐subject variables: drug (PLA, MPH, REB) and time (pre‐physical fatigue, post‐physical fatigue). During data processing and statistical modeling, pharmacological conditions were coded numerically (conditions 1–3) to maintain blinding of the investigators. Treatment labels were revealed after completion of all statistical analyses. ERP datasets were further stratified by component (N2, P3) and trial type (Go, No‐Go), with each combination analyzed separately to avoid violations of model assumptions caused by heterogeneous variance structures across cognitive processes (Volpert‐Esmond et al. 2018). Prior to modeling, descriptive statistics and visual inspection (histograms and boxplots) were used to evaluate distributional properties and identify potential outliers. No observations were excluded at the statistical stage.

#### 2.9.3 Data Analysis

Statistical analyses were performed in RStudio (version 4.4.1) using mixed effects models implemented in *lme4* (Douglas et al. 2015)*, lmertest* (Kuznetsova et al. 2017)*, glmmTMB* (Mollie et al. 2017) *emmeans* (Lenth 2024)*, Dharma* (Hartig 2024). “Participant” was included as a random effect to account for repeated measures and inter‐individual variability (Volpert‐Esmond et al. 2018; Vossen et al. 2011). The primary effect of interest was the drug × time interaction, which assessed whether pharmacological manipulation altered the impact of physical fatigue on cognitive performance. Behavioral measures of inhibitory control were the primary outcomes, whereas ERP measures were included as predefined secondary outcomes to investigate potential neurophysiological mechanisms underlying the observed behavioral effects. Go and No‐Go trials were analyzed separately because they reflect distinct cognitive processes (response execution and response inhibition, respectively) and are associated with different variance structures.

For behavioral outcomes, reaction time was analyzed using linear mixed‐effects models assuming Gaussian residuals. Accuracy values were analyzed using mixed‐effects beta regression models. Standard beta regression models were used for accuracy outcomes that fell within the open interval (0,1). For outcomes containing boundary values (observations exactly equal to 0 or 1), ordered beta mixed‐effects models were applied (Kubinec 2023), as these models accommodate boundary values without requiring transformation of the original data. To account for potential session effects associated with the crossover design, test session was included as a fixed factor in all behavioral models.

For neural outcomes, ERP amplitude (µV) and latency (ms) were analyzed using linear mixed‐effects models for each component (N2, P3) and electrode cluster (fronto‐central, centro‐parietal). To account for individual variability in physical fatigue‐related neural responses, models were first specified with random intercepts and random slopes for time within participant. When convergence issues or singular fits occurred, the correlation between the random slope and intercept was removed, and if required, the model was further simplified to include only a random intercept. All models were fitted using maximum likelihood estimation.

All model assumptions were evaluated using simulation‐based residual diagnostics (DHARMa), by inspecting residual uniformity, dispersion, and potential outliers. When violations were detected, the random‐effects structure was simplified or alternative model specifications were considered. When the interaction term was statistically significant, estimated marginal means were calculated and pairwise contrasts were performed using the Holm correction. Statistical significance was set at *p* = 0.05 (two‐tailed).

## 3 Results

To verify the effectiveness of the fatigue protocol, markers of physical fatigue were assessed during the experimental sessions. As previously reported (Arauz et al. 2026), the leg extension task reliably induced physical fatigue across all conditions, as reflected by increases in perceived fatigue and exertion, heart rate, and blood lactate (Supporting Information S2). Detailed descriptive statistics (Supporting Information S5), raw behavioral and fatigue data (Supporting Information S3), raw ERP data (Supporting Information S4), and full statistical model outputs for behavioral and neural outcomes (Supporting Information [Link], [Link]) are provided in the Supporting information S1: Materials.

### 3.1 Behavioral Performance

**FIGURE 3:**
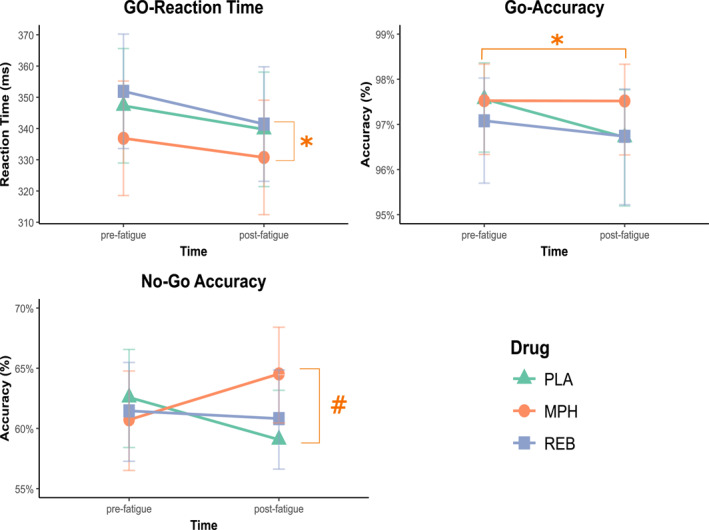
Behavioral performance across drug and physical fatigue. * Indicates a main effect of drug or time (*p* < 0.05) # indicates a significant MPH × time interaction (*p* < 0.05). MPH = methylphenidate; PLA = placebo; REB = reboxetine. Model‐estimated marginal means (±SE) are shown for go reaction time (top left), Go accuracy (top right), and No‐Go accuracy (bottom left) at pre‐ and post‐physical fatigue. Go reaction time showed a significant main effect of drug, with faster responses of MPH relative to placebo. Go accuracy revealed a significant main effect of time, reflecting lower accuracy following physical fatigue. No‐Go accuracy showed a significant MPH × Time interaction, reflecting differential pre–post changes of MPH relative to placebo. Together, these findings suggest that MPH altered behavioral responses to physical fatigue, particularly for inhibitory performance measured during No‐Go trials.

Behavioral performance on the Go/No‐Go task is shown in Figure 3. Session was included as a fixed factor to account for potential session effects associated with the crossover design. Significant effects of Session 2 relative to Session 1 were observed for No‐Go accuracy (OR = 1.19, 95% CI [1.06, 1.34], *p* = 0.002) and Go accuracy (OR = 1.24, 95% CI [1.04, 1.49], *p* = 0.019), whereas no session effects were observed for reaction time.

### 3.2 No‐Go Trials

No‐Go accuracy (% correct; mean ± SD) decreased from 62.56 ± 8.67% pre‐fatigue to 59.06 ± 8.67% post‐fatigue in the placebo condition, increased from 60.67 ± 8.39% to 63.67 ± 10.55% in the MPH condition, and remained largely unchanged in the REB condition (61.44 ± 7.88% to 60.78 ± 8.47%). Beta mixed‐effects analyses revealed no significant main effects of drug (MPH: OR = 0.92, 95% CI [0.78, 1.09], *p* = 0.346; REB: OR = 0.95, 95% CI [0.81, 1.12], *p* = 0.570), and the main effect of time did not reach statistical significance (OR = 0.86, 95% CI [0.73, 1.02], *p* = 0.076). However, a significant MPH × Time interaction was observed (OR = 1.37, 95% CI [1.09, 1.73], *p* = 0.008), indicating that the effect of physical fatigue differed in MPH relative to placebo. Holm‐adjusted within‐drug contrasts confirmed significantly higher No‐Go accuracy post‐fatigue compared with pre‐fatigue following MPH administration (OR = 0.85, SE = 0.07, *z* = −2.04, *p* = 0.042; Holm‐adjusted *p* = 0.048).

### 3.3 Go‐Trials

Mean Go reaction times (ms) before and after physical fatigue were 347.28 ± 42.67 and 339.73 ± 37.32 for placebo, 336.87 ± 32.12 and 330.74 ± 33.88 for MPH, and 351.91 ± 40.37 and 341.43 ± 36.87 for REB. Go accuracy (% correct; mean ± SD) was 97.28 ± 3.10% before fatigue and 96.33 ± 3.09% after fatigue in the placebo condition, 97.33 ± 2.99% and 97.39 ± 2.73% in the MPH condition, and 96.50 ± 3.42% and 96.33 ± 3.09% in the REB condition.

Linear mixed‐effects analyses revealed a significant main effect of drug on reaction time, with faster responses following MPH than placebo (*β* = −10.45, 95% CI [−18.96, −1.94], *p* = 0.018), whereas REB did not differ from placebo (*β* = 4.66, 95% CI [−3.86, 13.17], *p* = 0.287). The main effect of time was not significant (*β* = −7.55, 95% CI [−16.06, 0.96], *p* = 0.086), and no drug × time interactions were observed. For Go accuracy, a significant main effect of time indicated lower accuracy following physical fatigue (OR = 0.77, 95% CI [0.60, 0.99], *p* = 0.041). No significant main effects of drug or drug × time interactions were observed.

### 3.4 EEG‐ERP Components: N2 and P3

#### 3.4.1 No‐Go Trials

##### 3.4.1.1 Fronto‐Central Cluster (FC1, FC2)

**FIGURE 4:**
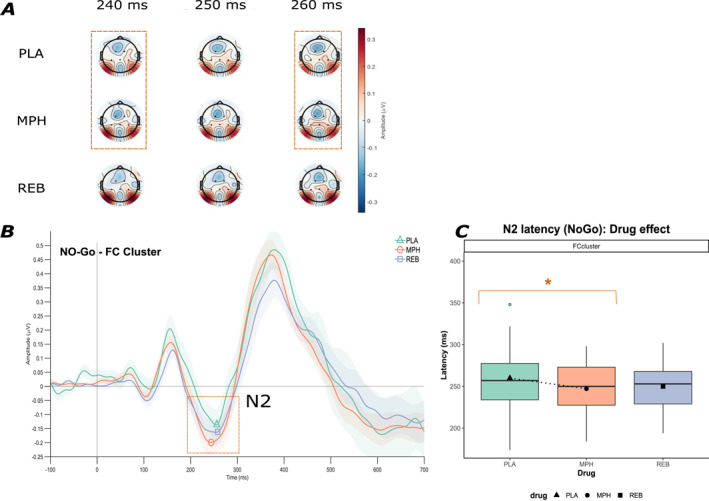
Main drug effect of No‐Go N2 latency at the fronto‐central cluster. * Indicates a significant main effect of MPH (*p* < 0.05). MPH = methylphenidate; PLA = placebo; REB = reboxetine. (A) Raw scalp topographies of the No‐Go condition around the N2 time window (240–260 ms) for the three drug conditions. The maps illustrate the characteristic fronto‐central distribution of the N2 component across conditions. While overall topographies are highly similar, a subtle temporal shift is observable: at 240 ms, the fronto‐central negative field appears slightly more centered for MPH, whereas for placebo the pattern is less well defined at this time point. By 260 ms, the topographies converge and appear highly comparable across conditions. Dashed boxes highlight the early (240 ms) and later (260 ms) time points used to illustrate this temporal convergence. (B) Grand‐average ERP waveforms for No‐Go trials at the fronto‐central (FC) cluster for the three drug conditions. The shaded area marks the N2 interval. The N2 peak occurs earlier of MPH compared with placebo and reboxetine, consistent with the observed reduction in fronto‐central N2 latency (C) boxplot of N2 latency at the FC cluster across drug conditions. MPH shows shorter N2 latencies relative to placebo, consistent with faster early conflict detection during response inhibition, whereas reboxetine does not show a comparable shift relative to placebo.

**FIGURE 5:**
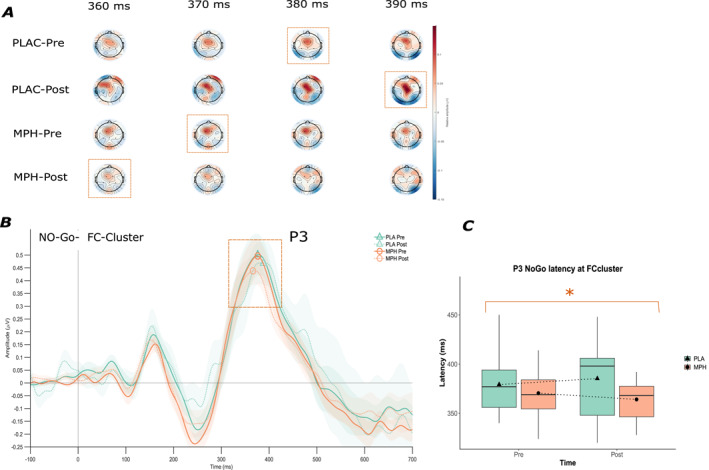
MPH × Time interaction of No‐Go P3 latency at the fronto‐central cluster. $ Indicates a significant MPH × time interaction (*p* < 0.05). MPH = methylphenidate; PLA = placebo; REB = reboxetine. (A) Scalp topographies illustrating the fronto‐central distribution of the No‐Go P3 component across time (360–390 ms) for each condition (PLA‐Pre, PLA‐Post, MPH‐Pre, MPH‐Post). Topographies are shown relative to a baseline defined as the average of the first two time points within each condition, such that colors reflect deviations from this early interval rather than absolute amplitudes. The highlighted maps indicate the approximate peak of the P3 component within each condition. (B) Grand‐average ERP waveforms extracted from the fronto‐central cluster (FC1, FC2) for No‐Go trials. The shaded region marks the P3 time window used for latency estimation. Waveforms show that the timing of the P3 peak differs between drug and physical fatigue conditions. (C) Boxplots of P3 latency for No‐Go trials within the fronto‐central cluster. A significant drug × time interaction was observed for fronto‐central No‐Go P3 latency, indicating differential fatigue‐related changes across pharmacological conditions.

No significant main effects of drug or time, and no drug × time interactions were observed for fronto‐central N2 or P3 amplitude. For N2 latency, a significant main effect of drug was observed, with shorter latencies for MPH relative to placebo (*β* = −13.00, 95% CI [−25.11, −0.89], *p* = 0.038; Figure 4). No significant main effect of time was observed (*β* = 1.89, 95% CI [−10.22, 14.00], *p* = 0.761), and no significant drug × time interactions were detected. For P3 latency, no significant main effects of drug or time were observed. However, a significant MPH × Time interaction was observed (*β* = −20.22, 95% CI [−39.88, −0.56], *p* = 0.047; Figure 5), indicating that fatigue‐related changes in P3 latency differed between MPH and placebo. Holm‐adjusted post hoc comparisons revealed no significant pre–post differences within placebo (*p* = 0.060), MPH (*p* = 0.388), or REB (*p* = 0.832).

##### 3.4.1.2 Centro‐Parietal Cluster (CPz, CP3, CP4)

No significant main effects of drug or time, and no drug × time interactions, were observed for N2 or P3 amplitude or latency.

#### 3.4.2 Go Trials

##### 3.4.2.1 Fronto‐Central Cluster (FC1, FC2)

No significant main effects of drug or time, and no drug × time interactions, were observed for N2 or P3 amplitude or latency.

##### 3.4.2.2 Centro‐Parietal Cluster (CPz, CP3, CP4)

For amplitude, no significant effects were observed for N2. In contrast, P3 amplitude showed a significant main effect of time (*β* = −0.10 μV, 95% CI [−0.18, −0.01], *p* = 0.031), indicating reduced P3 amplitude following physical fatigue. No significant main effect of drug or drug × time interactions were observed (MPH × Time: *β* = 0.11 μV, 95% CI [−0.01, 0.23], *p* = 0.086; REB × Time: *β* = 0.08 μV, 95% CI [−0.04, 0.20], *p* = 0.218). No significant effects were observed for N2 or P3 latency.

## 4 Discussion

The present study investigated whether catecholaminergic modulation influenced inhibitory control following acute physical fatigue. Participants showed improved No‐Go accuracy following physical fatigue when receiving MPH, whereas no comparable changes were observed following placebo or REB administration. ERP analyses revealed MPH‐related effects on fronto‐central No‐Go latency measures. Together, these findings suggest that both dopaminergic and noradrenergic systems contribute to inhibitory control following physical fatigue and provide preliminary neurophysiological evidence that these effects may be accompanied by alterations in the temporal dynamics of inhibitory processing.

### 4.1 Behavioral Findings

A pharmacological effect was observed for No‐Go accuracy, with improved inhibitory performance following physical fatigue in the MPH condition. This finding suggests that catecholaminergic modulation may facilitate inhibitory control following fatigue. Specifically, MPH may have supported the functioning of neural networks involved in inhibitory control through its broader catecholaminergic actions (Linssen et al. 2014; Arnsten 2011), thereby contributing to improved cognitive performance after physical fatigue. This interpretation aligns with cognitive–energetic models proposing that executive performance depends on an optimal catecholaminergic state (Cools and D'Esposito 2011), whereas deviations from this optimum may impair performance (McMorris et al. 2011; Sanders 1983).

The absence of comparable effects of REB suggests that selective noradrenergic reuptake inhibition alone may be insufficient to maintain inhibitory control following physical fatigue. Importantly, this finding does not imply that noradrenergic mechanisms are unimportant. Evidence indicates that DA and NA interact to support prefrontal cognitive function (Ranjbar‐Slamloo and Fazlali 2020; Xing et al. 2016), and the differential effects of MPH and REB suggest that successful maintenance of inhibitory control may depend on the combined modulation of these catecholaminergic systems rather than on noradrenergic enhancement alone. Recent neuroimaging evidence has shown that MPH‐induced changes in large‐scale brain organization are associated with regional NA transporter (NET) density (Tomasi et al. 2025). This is consistent with the role of NET in regulating cortical DA availability, particularly in the prefrontal cortex where DA transporter (DAT) density is low and NET contributes to DA clearance (Xing et al. 2016; Aggarwal and Mortensen 2017). In contrast, DAT density is considerably higher in striatal regions (Aggarwal and Mortensen 2017), allowing MPH, but not REB, to influence both dopaminergic and noradrenergic signaling across a broader set of neural systems. Together, these findings suggest that the beneficial effects of MPH are unlikely to be attributable to either neurotransmitter system in isolation, but instead reflect integrated catecholaminergic modulation of neural networks supporting cognitive control. Accordingly, the present findings extend current understanding of catecholaminergic contributions to cognitive control (Ranjbar‐Slamloo and Fazlali 2020; Xing et al. 2016), by demonstrating their relevance under conditions of physical fatigue, in which maintaining effective inhibitory control may be particularly challenging.

MPH was also associated with faster Go responses irrespective of fatigue state, indicating a more general effect on task performance. This finding is consistent with previous evidence showing that MPH enhances processing speed and attention (Linssen et al. 2014). Nevertheless, the most prominent pharmacological differences following physical fatigue were observed for No‐Go measures, suggesting that catecholaminergic modulation may be particularly relevant for maintaining inhibitory control under conditions of physical fatigue.

### 4.2 Neural Findings

ERP analyses were included as predefined secondary outcomes to investigate potential neurophysiological mechanisms underlying the behavioral effects. A significant MPH × Time interaction was observed for fronto‐central No‐Go P3 latency, indicating that the effects of physical fatigue on the later stages of inhibitory processing and stimulus evaluation (Huster et al. 2013) differed between MPH and placebo. However, Holm‐adjusted post hoc comparisons did not reveal significant pre‐post differences within placebo, MPH, or REB. Consequently, the functional significance of this interaction remains unclear and should be interpreted cautiously.

In addition to this fatigue‐related effect, MPH was associated with shorter fronto‐central No‐Go N2 latencies irrespective of fatigue state. The N2 component has been linked to conflict monitoring and the early stages of inhibitory processing (Huster et al. 2013). Earlier N2 latencies may reflect earlier engagement of neural processes involved in conflict detection and the initiation of cognitive control.

In contrast to our hypothesis, pharmacological modulation did not influence P3 amplitude. The absence of amplitude effects suggests that MPH did not primarily alter the magnitude of neural activity associated with inhibitory processing. Rather, together with the observed N2 and P3 latency effects, the findings indicate that catecholaminergic modulation may have influenced the temporal dynamics of neural processes supporting inhibitory control. This interpretation is consistent with previous work highlighting the importance of neural timing for successful response inhibition (Hervault et al. 2025). The localization of these effects to the canonical fronto‐central scalp distribution of inhibitory‐control‐related activity further supports their relevance to inhibitory processing. Although the relationship between the observed ERP and behavioral effects remains unclear, the present findings suggest that catecholaminergic modulation may influence the temporal dynamics of neural processes involved in inhibitory control.

### 4.3 Integration With Previous Findings

The present findings extend previous analyses from the same randomized crossover trial, which examined the effects of catecholaminergic modulation on physical performance and fatigue perception (Arauz et al. 2026). In that study, MPH increased alertness, vigor, and perceived performance without enhancing task output, whereas REB reduced physical performance with minimal perceptual change. In the present study, MPH was associated with improved inhibitory performance, whereas REB did not influence cognitive control measures. Together, these findings suggest that catecholaminergic modulation may differentially influence the cognitive, perceptual, and physical consequences of physical fatigue. Notably, MPH was associated with improvements in both subjective responses to fatigue and cognitive performance, whereas REB did not produce comparable cognitive benefits. Although the mechanisms underlying these effects cannot be determined from the present data, the findings suggest that the catecholaminergic mechanisms supporting cognitive responses to fatigue may differ, at least in part, from those regulating physical performance during sustained exertion (Klass et al. 2012, 2016, 2018). Collectively, these findings support the view that the cognitive and physical consequences of fatigue are mediated by partially distinct catecholaminergic processes (Roelands and Meeusen 2010; Meeusen and Roelands 2010).

### 4.4 Limitations and Future Directions

Several limitations should be considered when interpreting the present findings. First, although the randomized crossover design increased statistical efficiency, the sample size was modest. While the study was adequately powered for the primary outcome and robustly accounted for subject‐ and time‐related variability, it may have had limited sensitivity to detect smaller pharmacological effects, particularly for ERP outcomes and interaction effects. Consequently, the ERP findings should be considered preliminary and require replication in larger independent samples. Furthermore, the sample size precluded investigation of potential sex differences, despite evidence that both catecholaminergic function (Becker et al. 2017) and responses to physical fatigue (Hunter 2014) may vary between males and females. Second, the pharmacological agents primarily target dopaminergic and noradrenergic reuptake but may also exert indirect effects on other monoaminergic systems (Miller et al. 2002; King et al. 2018), which should be considered when interpreting the findings. In addition, individual variability in pharmacological responsiveness may have contributed to the observed variability in treatment effects (Linssen et al. 2014). Finally, the present study focused on inhibitory control using a Go/No‐Go paradigm and employed a localized dynamic resistance exercise protocol to induce fatigue. While this approach provides substantial experimental control and reliably elicits muscular fatigue, the resulting physiological and neurocognitive responses may not fully generalize to whole‐body endurance exercise such as running or cycling. Whole‐body exercise imposes additional cardiovascular, metabolic, and thermoregulatory demands (Meeusen et al. 2021) that may influence cognitive performance through partially distinct mechanisms. Establishing the ecological validity of these effects will be important for understanding the extent to which catecholaminergic modulation influences cognitive performance during physical fatigue in real‐world settings. Despite these limitations, the present study offers novel mechanistic insights into the role of catecholaminergic systems in inhibitory control following physical fatigue.

## 5 Conclusion

The present study provides novel pharmacological evidence that catecholaminergic mechanisms contribute to inhibitory control following physical fatigue. MPH was associated with improved No‐Go accuracy following physical fatigue, whereas REB did not produce comparable behavioral effects. At the neural level, MPH was associated with alterations in inhibitory‐control‐related ERP latency measures, suggesting potential changes in the temporal dynamics of inhibitory processing. Together, the findings provide mechanistic insights into the role of catecholaminergic neurotransmission in maintaining inhibitory control following physical fatigue.

## Author Contributions

Conceptualization was conducted by Y.L.A.A., K.D.P., B.R. and U.M. Funding acquisition was managed by B.R., and U.M. Resources were provided by B.R. and K.D.P. Project administration was led by Y.L.A.A. Methodology was developed by Y.L.A.A., K.D.P., B.R. and U.M. Investigation and data curation were carried out by Y.L.A.A. Formal analysis and visualization were performed by Y.L.A.A. and N.O. The original draft of the manuscript was written by Y.L.A.A., with review and editing contributions from N.O., M.A.P.D., B.V.B., J.H., K.D.P., B.R. and U.M. Supervision was provided by U.M., B.R., and K.D.P. All authors reviewed and approved the final manuscript. All individuals listed as authors meet the criteria for authorship, and all who qualify have been included.

## Funding

Y. Laurisa Arenales Arauz is a recipient of a doctoral grant funded by FWO_WEAVE (G095422N). Bart Roelands and Kevin De Pauw are members of the Strategic Research Program Exercise and the Brain in Health & Disease: The Added Value of Human‐Centered Robotics (SRP77). Jelle Habay is a recipient of a fundamental aspirant fellowship funded by the Research Foundation Flanders (FWO) (project number: 11J6323N). U.M. would like to acknowledge financial support from the Slovenian Research and Innovation Agency (ARIS): Research Program Kinesiology for Quality of Life under Grant P5‐0381 as well as the funding under the Horizon Europe—TBrainBoost, grant agreement No. 101120150.

## Ethics Statement

This study was approved by the medical ethics commission at the UZ Brussel (protocol: 2022‐002836‐30) on 15/03/2023 in accordance with the declaration of Helsinki. Informed consent was obtained from the participants.

## Consent

All participants gave written informed consent to participate in the present study.

## Conflicts of Interest

The authors declare no conflicts of interest.

## Permission to Reproduce Material From Other Sources

The authors have nothing to report.

## Supporting information


Supporting Information S1

Supporting Information S2

Supporting Information S3

Supporting Information S4

Supporting Information S5

Supporting Information S6

Supporting Information S7

## Data Availability

All raw data collected during this study are included in Supporting Information S3 and S4.
