# Supplementary material for: Catecholaminergic Contributions to Inhibitory Control Following Physical Fatigue: Behavioral and Neurophysiological Findings
Source: Eur J Sport Sci. 2026 Aug 17;26(9):e70231. doi: 10.1002/ejsc.70231 (PMC13481479; doi:10.1002/ejsc.70231)
Supplement: Supplementary file 1 [file EJSC-26-e70231-s001.docx]

| **Supplementary File 7. Model outputs for neural outcomes.** Full statistical model outputs for ERP measures, including parameter estimates, confidence intervals, and model diagnostics. | | | | | |
| --- | --- | --- | --- | --- | --- |
| **Outcome** | **Effect** | **β [95% CI]** | | **p value** | |
|  |  | **N2** | **P3** | **N2** | **P3** |
| **GO-trials** |  |  | |  | |
| - **Amplitude** (µV) |  |  |  |  |  |
| - **FCcluster** | Intercept | -0.22 [0.29, -0.16] | -0.24 [-0.29, -0.16] | / | / |
|  | Time (Post vs Pre) | 0.05 [-0.009, -0.11] | 0.06 [-0.01, 0.11] | 0.099 | 0.869 |
|  | Drug (MPH vs PLA) | -0.02 [-0.08, 0.04] | -0.03 [-0.08, 0.04] | 0.444 | 0.139 |
|  | Drug (REB vs PLA) | 0.00 [-0.05, 0.06] | -0.01 [-0.05, 0.06] | 0.889 | 0.504 |
|  | MPH x Time | -0.01 [-0.10, 0.07] | 0.03 [-0.07, 0.14] | 0.723 | 0.555 |
|  | REB x Time | -0.01 [-0.09, 0.08] | 0.03 [-0.08, 0.14] | 0.869 | 0.578 |
| - **CPcluster** | Intercept | -0.27 [-0.37, -0.17] | 0.48 [0.37, 0.60] | / | / |
|  | Time (Post vs Pre) | 0.01 [-0.04, 0.07] | -0.10 [-0.18, -0.01] | 0.873 | **0.031*** |
|  | Drug (MPH vs PLA) | 0.00 [-0.06, 0.05] | 0.03 [-0.06, 0.12] | 0.762 | 0.497 |
|  | Drug (REB vs PLA) | 0.01 [-0.04, 0.06] | -0.02 [-0.11, 0.06] | 0.671 | 0.621 |
|  | MPH x Time | 0.03 [-0.04, 0.10] | 0.11 [-0.01, 0.23] | 0.438 | 0.086 |
|  | REB x Time | 0.03 [-0.05, 0.10] | 0.08 [-0.04, 0.20] | 0.501 | 0.218 |
| - **Latency (ms)** |  |  | |  | |
| - **FCcluster** | Intercept | 254.67 [242.54, 266.79] | 377.56 [356.63, 398.48] | / | / |
|  | Time (Post vs Pre) | -5.44 [-11.57, 0.68] | 4.33 [-17.18, 25.85] | 0.086 | 0.694 |
|  | Drug (MPH vs PLA) | -2.00 [-7.98, 3.98] | 4.89 [-16.62, 26.40] | 0.514 | 0.657 |
|  | Drug (REB vs PLA) | 0.56 [-5.42, 6.53] | 11.89 [-9.62, 33.40] | 0.865 | 0.282 |
|  | MPH x Time | 5.33 [-3.12, 13.79] | -11.56 [-41.98, 18.87] | 0.220 | 0.458 |
|  | REB x Time | 0.67 [-7.79, 9.12] | -1.11 [-31.53, 29.31] | 0.878 | 0.943 |
| - **CPcluster** | Intercept | 168.56 [154.72, 182.39] | 303.67 [284.74, 322.59] | / | / |
|  | Time (Post vs Pre) | -1.00 [-8.08, 6.08] | -9.67 [-26.82, 7.48] | 0.783 | 0.272 |
|  | Drug (MPH vs PLA) | -0.78 [-7.86, 6.30] | 8.00 [-9.15, 25.15] | 0.830 | 0.363 |
|  | Drug (REB vs PLA) | 3.33 [-3.75, 10.41] | 0.67 [-16.48, 17.82] | 0.359 | 0.939 |
|  | MPH x Time | -3.89 [-13.90, 6.12] | 0.33 [-23.92, 24.58] | 0.449 | 0.979 |
|  | REB x Time | 1.33 [-8.68, 11.34] | 6.11 [-18.14, 30.36] | 0.795 | 0.623 |
| **NO-GO-trials** |  |  |  |  |  |
| - **Amplitude** (µV) |  |  |  |  |  |
| - **FCcluster** | Intercept | -0.26 [-0.36, -0.16] | 0.59 [0.44, 0.74] | / | / |
|  | Time (Post vs Pre) | 0.02 [-0.08, 0.11] | 0.12 [-0.03, 0.26] | 0.748 | 0.117 |
|  | Drug (MPH vs PLA) | -0.05 [-0.14, 0.05] | -0.03 [-0.16, 0.11] | 0.322 | 0.703 |
|  | Drug (REB vs PLA) | 0.00 [-0.10, 0.09] | -0.12 [-0.26, 0.01] | 0.969 | 0.083 |
|  | MPH x Time | -0.01 [-0.15, 0.12] | -0.16 [-0.36, 0.03] | 0.878 | 0.102 |
|  | REB x Time | 0.01 [-0.14, 0.13] | -0.15 [-0.34, 0.05] | 0.927 | 0.145 |
| - **CPcluster** | Intercept | -0.19 [-0.30, -0.07] | 0.37 [0.23, 0.50] | / | / |
|  | Time (Post vs Pre) | -0.07 [-0.18, 0.04] | -0.08 [-0.23, 0.07] | 0.219 | 0.292 |
|  | Drug (MPH vs PLA) | 0.02 [-0.09, 0.13] | 0.04 [-0.11, 0.19] | 0.718 | 0.594 |
|  | Drug (REB vs PLA) | -0.04 [-0.15, 0.07] | -0.06 [-0.21, 0.09] | 0.455 | 0.424 |
|  | MPH x Time | 0.07 [-0.08, 0.23] | 0.04 [-0.17, 0.24] | 0.353 | 0.736 |
|  | REB x Time | 0.09 [-0.07, 0.24] | 0.06 [-0.15, 0.26] | 0.288 | 0.593 |
| - **Latency (ms)** |  |  |  |  |  |
| - **FCcluster** | Intercept | 259.00 [244.05, 273.95] | 379.22 [363.69, 394.76] | / | / |
|  | Time (Post vs Pre) | 1.89 [-10.22, 14.00] | 13.89 [-0.01, 27.79] | 0.761 | 0.053 |
|  | Drug (MPH vs PLA) | -13.00 [-25.11, -0.89] | -8.78 [-22.68, 5.12] | **0.038*** | 0.219 |
|  | Drug (REB vs PLA) | -7.00 [-19.11, 5.11] | 7.67 [-6.23, 21.57] | 0.260 | 0.283 |
|  | MPH x Time | 0.67 [-16.46, 17.79] | -20.22 [-39.88, -0.56] | 0.939 | **0.047*** |
|  | REB x Time | -5.78 [-22.90, 11.34] | -12.33 [-31.99, 7.33] | 0.510 | 0.222 |
| - **CPcluster** | Intercept | 191.56 [166.32, 216.79] | 324.33 [295.22, 353.44] | / | / |
|  | Time (Post vs Pre) | -1.89 [-17.26, 13.48] | -6.89 [-27.74, 13.96] | 0.810 | 0.519 |
|  | Drug (MPH vs PLA) | -4.00 [-19.15, 11.15] | -5.00 [-25.86, 15.86] | 0.606 | 0.640 |
|  | Drug (REB vs PLA) | 4.11 [-11.04, 19.26] | -5.11 [-25.97, 15.75] | 0.596 | 0.632 |
|  | MPH x Time | 8.22 [-13.20, 29.64] | -0.22 [-29.72, 29.28] | 0.454 | 0.988 |
|  | REB x Time | -11.89 [-33.31, 9.53] | 1.44 [-28.06, 30.95] | 0.280 | 0.924 |

^* p<0.05^
**^Note.^** ^β = regression coefficient; CI = confidence interval. PLA = placebo; MPH = methylphenidate; REB = reboxetine. IRR = incidence rate ratio. FC cluster = fronto-central electrode cluster (FC1, FC2); CP cluster = centro-parietal electrode cluster (CPz, CP3, CP4). N2 and P3 refer to ERP components.^
