# Supplementary material for: Catecholaminergic Contributions to Inhibitory Control Following Physical Fatigue: Behavioral and Neurophysiological Findings
Source: Eur J Sport Sci. 2026 Aug 17;26(9):e70231. doi: 10.1002/ejsc.70231 (PMC13481479; doi:10.1002/ejsc.70231)
Supplement: Supplementary file 2 [file EJSC-26-e70231-s002.docx]

| **Supplementary File 6. Model outputs for behavioral outcomes.** Full statistical model outputs for behavioral measures (e.g., Go/No-Go performance), including parameter estimates, confidence intervals, and model diagnostics. | | | |
| --- | --- | --- | --- |
| **Outcome** | **Effect** | **β/OR [95% CI]** | **p** |
| **Go – Reaction Time (ms)** | Intercept | 346.56 [328.93, 364.19] | / |
|  | Time (Post vs Pre) | -7.55 [-16.06, 0.96] | 0.086 |
|  | Drug (MPH vs PLA) | -10.45 [-18.96, -1.94] | **0.018*** |
|  | Drug (REB vs PLA) | 4.66 [-3.86, 13.17] | 0.287 |
|  | Session 2 vs 1 | 0.63 [-5.42, 6.69] | 0.838 |
|  | Session 3 vs 1 | 1.54 [-4.48, 7.57] | 0.617 |
|  | MPH x Time | 1.42 [-10.61, 13.45] | 0.818 |
|  | REB x Time | -2.93 [-14.96, 9.10] | 0.634 |
| **Go – Accuracy (0-1)** | Intercept | OR: 38.9 [25.19, 60.08] | / |
|  | Time (Post vs Pre) | OR: 0.77 [0.60, 0.99] | **0.041*** |
|  | Drug (MPH vs PLA) | OR: 1.02 [0.78, 1.34] | 0.880 |
|  | Drug (REB vs PLA) | OR: 0.89 [0.68, 1.15] | 0.356 |
|  | MPH x Time | OR: 1.33 [0.92, 1.91] | 0.130 |
|  | REB x Time | OR: 1.17 [0.83, 1.65] | 0.375 |
|  | Session 2 vs 1 | OR: 1.24 [1.04, 1.49] | **0.019*** |
|  | Session 3 vs 1 | OR: 1.00 [0.84, 1.18] | 0.972 |
| **No-Go- Accuracy (0-1)** | Intercept | OR 1.67 [1.40, 1.99] | / |
|  | Time (Post vs Pre) | OR: 0.86 [0.73, 1.02] | 0.076 |
|  | Drug (MPH vs PLA) | OR: 0.92 [0.78, 1.09] | 0.346 |
|  | Drug (REB vs PLA) | OR: 0.95 [0.81, 1.12] | 0.570 |
|  | Session 2 vs 1 | OR: 1.19 [1.06, 1.34] | **0.002*** |
|  | Session 3 vs 1 | OR: 1.10 [0.99, 1.24] | 0.084 |
|  | MPH x Time**^1^** | OR: 1.37 [1.09, 1.73] | **0.008*** |
|  | REB x Time | OR: 1.13 [0.89, 1.43] | 0.305 |

**p* < 0.05.
^1^Holm-corrected post hoc comparison showed lower accuracy post-fatigue compared to pre-fatigue under MPH conditions (OR = 0.85, SE = 0.07, *z* = −1.98, *p* = 0.048). **Note.** Abbreviations: PLA = placebo; MPH = methylphenidate; REB = reboxetine; OR = incidence rate ratio.
