# Supplementary material for: Catecholaminergic Contributions to Inhibitory Control Following Physical Fatigue: Behavioral and Neurophysiological Findings
Source: Eur J Sport Sci. 2026 Aug 17;26(9):e70231. doi: 10.1002/ejsc.70231 (PMC13481479; doi:10.1002/ejsc.70231)
Supplement: Supplementary file 3 [file EJSC-26-e70231-s004.docx]

| **Supplementary File 5. Descriptive statistics for behavioral and neural outcomes.**  Summary statistics (means, standard deviations, and distributions) for all behavioral and ERP measures across conditions and time points. | | | | | | | |
| --- | --- | --- | --- | --- | --- | --- | --- |
| **Outcomes** | **Descriptives**  behavioural: (mean ± std ) / ERPs: (mean ± SE) | | | | | | |
|  | **Time** | **PLA** | | **MPH** | | **REB** | |
| **Behavioural performance** |  |  | | | | | |
| Go – Reaction Time (ms) | *Pre* | 347.28 ± 42.67 | | 336.87 ±32.12 | | 351.91 ± 40.37 | |
|  | *Post* | 339.73 ± 37.32 | | 330.74 ± 33.88 | | 341.43 ± 36.87 | |
| Go – Accuracy (%) | *Pre* | 97.28 ± 3.10 | | 97.33 ± 2.99 | | 96.50 ± 3.42 | |
|  | *Post* | 96.33 ± 3.09 | | 97.39 ± 2.73 | | 96.33 ± 3.09 | |
| No-Go- Accuracy (%) | *Pre* | 62.56 ± 8.67 | | 60.67 ± 8.39 | | 61.44 ± 7.88 | |
|  | *Post* | 59.06 ± 8.67 | | 63.67 ± 10.55 | | 60.78 ± 8.47 | |
| **ERPs** |  |  | | | | | |
| **GO- trials** |  | **N2** | **P3** | **N2** | **P3** | **N2** | **P3** |
| - Amplitude(µV) |  |  | |  | |  | |
| - FCcluster | *Pre* | -0.22 ± 0.02 | 0.23 ± 0.04 | -0.24 ± 0.14 | 0.18 ± 0.03 | -0.21 ± 0.03 | 0.21 ± 0.03 |
|  | *Post* | -0.25 ± 0.06 | -0.17 ± 0.11 | -0.21± 0.04 | 0.20 ± 0.05 | -0.17 ± 0.02 | 0.23 ±0.04 |
| - CPcluster | *Pre* | -0.26 ± 0.05 | 0.48 ± 0.05 | -0.27 ± 0.03 | 0.51 ± 0.05 | -0.26 ± 0.05 | 0.46 ± 0.07 |
|  | *Post* | 0.38 ± 0.06 | 0.39 ± 0.06 | -0.23 ± 0.06 | 0.52 ±0.06 | -0.22 ± 0.06 | 0.44 ± 0.04 |
| - Latency (ms) |  |  | |  | |  | |
| - FCcluster | *Pre* | 254.67 ± 6.01 | 377.56 ± 10.60 | 252.66 ± 6.50 | 381.44 ± 13.81 | 255.22 ± 6.2 | 389.44 ± 8.90 |
|  | *Post* | 249.22 ± 6.44 | 381.89 ± 12.67 | 252.55 ± 6.62 | 375.22 ± 7.28 | 172.22 ± 6.69 | 392.67 ± 11.29 |
| - CPcluster | *Pre* | 168.55 ± 7.38 | 303.67 ± 10.04 | 167.77 ± 7.64 | 311.67 ± 11.51 | 171.89 ± 7.10 | 304.33 ± 8.92 |
|  | *Post* | 167.56 ± 7.40 | 294.0 ± 7.68 | 162.89 ± 7.32 | 302.33 ± 11.19 | 250.44 ±7.44 | 300.78 ± 9.71 |
| **No-Go- trials** |  |  | |  | |  | |
| - Amplitude(µV) |  |  | |  | |  | |
| - FCcluster | *Pre* | -0.26 ± 0.04 | 0.58 ± 0.08 | -0.31 ± 0.04 | 0.56 ± 0.08 | -0.26 ± 0.05 | 0.46 ± 0.05 |
|  | *Post* | -0.24 ± 0.07 | 0.70 ± 0.08 | -0.30 ± 0.05 | 0.51 ± 0.06 | -0.25 ± 0.04 | 0.43 ± 0.07 |
| - CPcluster | *Pre* | -0.18 ± 0.05 | 0.36 ± 0.08 | -0.17 ± 0.05 | 0.40 ± 0.05 | -0.22 ± 0.05 | 0.30 ± 0.06 |
|  | *Post* | 0.25 ± 0.08 | 0.28 ± 0.09 | -0.16 ±0.05 | 0.36 ± 0.07 | 0.21 ±0.06 | 0.28 ± 0.07 |
| - Latency (ms) |  |  | |  | |  | |
| - FCcluster | *Pre* | 259.00 ± 5.31 | 379.22 ± 7.12 | 246.00 ± 6.83 | 370.44 ± 5.36 | 252.00 ± 5.41 | 386.88 ± 8.52 |
|  | *Post* | 260.89 ± 11.48 | 393.11 ± 12.04 | 248.55 ± 8.03 | 364.11 ± 4.66 | 248.11 ± 8.22 | 388.44 ± 8.90 |
| - CPcluster | *Pre* | 191.55 ± 13.01 | 324.33 ± 15.08 | 187.55 ± 13.35 | 319.33 ± 14.06 | 195.67 ± 12.19 | 319.22 ± 12.92 |
|  | *Post* | 189.67 ± 13.76 | 317.44 ± 15.96 | 193.88 ±14.88 | 312.22 ± 14.83 | 181.89 ± 12.07 | 313.77 ± 18.28 |

*Note. PLA = placebo; MPH = methylphenidate; REB = reboxetine. FC cluster = fronto-central electrode cluster (FC1, FC2); CP cluster = centro-parietal electrode cluster (CPz, CP3, CP4).*
