# Supplementary material for: Catecholaminergic Contributions to Inhibitory Control Following Physical Fatigue: Behavioral and Neurophysiological Findings
Source: Eur J Sport Sci. 2026 Aug 17;26(9):e70231. doi: 10.1002/ejsc.70231 (PMC13481479; doi:10.1002/ejsc.70231)
Supplement: Supplementary file 4 [file EJSC-26-e70231-s005.docx]

**Supplementary File 1. Verification of physical fatigue protocol.**
Summary data demonstrating the effectiveness of the fatigue protocol, including perceived fatigue and exertion (A,B), heart rate (C), and blood lactate (D) levels across experimental conditions.

**Supplementary File 2. Raw behavioral and physical fatigue dataset.**
Complete individual-level data for Go/No-Go task performance and physical fatigue markers (perceived fatigue, exertion, heart rate, and blood lactate) for all participants across conditions.

**Supplementary File 3. Raw Event Related Potentials (ERP) dataset.**
Individual-level ERP data for all participants, including neural measures (amplitude and latency) derived from the Go/No-Go task across experimental conditions.

**Supplementary File 4. Descriptive statistics for behavioral and neural outcomes.**
Summary statistics (means, standard deviations, and distributions) for all behavioral and ERP measures across conditions and time points.

**Supplementary File 5. Model outputs for behavioral outcomes.**
Full statistical model outputs for behavioral measures (e.g., Go/No-Go performance), including parameter estimates, confidence intervals, and model diagnostics.

**Supplementary File 6. Model outputs for neural outcomes.**
Full statistical model outputs for ERP measures, including parameter estimates, confidence intervals, and model diagnostics.
