# Supplementary material for: Catecholaminergic Contributions to Inhibitory Control Following Physical Fatigue: Behavioral and Neurophysiological Findings
Source: Eur J Sport Sci. 2026 Aug 17;26(9):e70231. doi: 10.1002/ejsc.70231 (PMC13481479; doi:10.1002/ejsc.70231)
Supplement: Supplementary file 5 [file EJSC-26-e70231-s007.docx]

**

**

**C.**

*

**D.**

*

* p < 0.05

** p < 0.001

Supplementary File 2. Verification of physical fatigue protocol.

Summary data demonstrating the effectiveness of the fatigue protocol, including perceived

fatigue and exertion (A,B), heart rate (C), and blood lactate (D) levels across experimental conditions.
